# Resveratrol production from several types of saccharide sources by a recombinant *Scheffersomyces stipitis* strain

**DOI:** 10.1016/j.mec.2021.e00188

**Published:** 2021-11-26

**Authors:** Yuma Kobayashi, Kentaro Inokuma, Mami Matsuda, Akihiko Kondo, Tomohisa Hasunuma

**Affiliations:** a Graduate School of Science, Technology and Innovation, Kobe University, 1-1 Rokkodai-cho, Nada-ku, Kobe, 657-8501, Japan; b Engineering Biology Research Center, Kobe University, 1-1 Rokkodai-cho, Nada-ku, Kobe, 657-8501, Japan; c Biomass Engineering Program, RIKEN, 1-7-22 Suehiro-cho, Tsurumi-ku, Yokohama, Kanagawa, 230-0045, Japan

**Keywords:** *Scheffersomyces stipitis*, Non-conventional yeast, Resveratrol, Metabolic engineering, Shikimate pathway, Disaccharide dissimilation

## Abstract

Resveratrol is a plant-derived aromatic compound with a wide range of beneficial properties including antioxidant and anti-aging effects. The resveratrol currently available on the market is predominantly extracted from certain plants such as grape and the Japanese knotweed *Polygonum cuspidatum*. Due to the unstable harvest of these plants and the low resveratrol purity obtained, it is necessary to develop a stable production process of high-purity resveratrol from inexpensive feedstocks. Here, we attempted to produce resveratrol from a wide range of sugars as carbon sources by a using the genetically-engineered yeast *Scheffersomyces stipitis* (formerly known as *Pichia stipitis*), which possesses a broad sugar utilization capacity. First, we constructed the resveratrol producing strain by introducing genes coding the essential enzymes for resveratrol biosynthesis [tyrosine ammonia-lyase 1 derived from *Herpetosiphon aurantiacus* (*HaTAL1*), 4-coumarate: CoA ligase 2 derived from *Arabidopsis thaliana* (*At4CL2*), and stilbene synthase 1 derived from *Vitis vinifera* (*VvVST1*)]. Subsequently, a feedback-insensitive allele of chorismate mutase was overexpressed in the constructed strain to improve resveratrol production. The constructed strain successfully produced resveratrol from a broad range of biomass-derived sugars [glucose, fructose, xylose, *N*-acetyl glucosamine (GlcNAc), galactose, cellobiose, maltose, and sucrose] in shake flask cultivation. Significant resveratrol titers were detected in cellobiose and sucrose fermentation (529.8 and 668.6 mg/L after 120 h fermentation, respectively), twice above the amount obtained with glucose (237.6 mg/L). Metabolomic analysis revealed an altered profile of the metabolites involved in the glycolysis and shikimate pathways, and also of cofactors and metabolites of energy metabolisms, depending on the substrate used. The levels of resveratrol precursors such as L-tyrosine increased in cellobiose and sucrose-grown cells. The results indicate that *S. stipitis* is an attractive microbial platform for resveratrol production from broad types of biomass-derived sugars and the selection of suitable substrates is crucial for improving resveratrol productivity of this yeast.

## Highlights

• A recombinant *S. stipitis* strain for resveratrol production was constructed.
• The constructed strain produced resveratrol from a broad range of sugars.
• High resveratrol titers were observed in fermentation with cellobiose and sucrose.
• Carbon source affected intracellular accumulation of some resveratrol precursors.

## Graphical abstract

**Figure:**
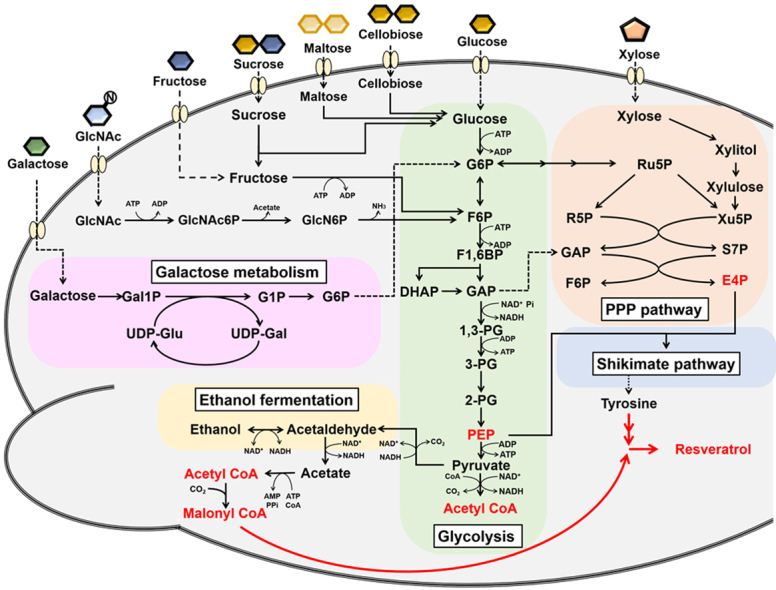

## 1 Introduction

Resveratrol (3,5,4′-trihydroxystilbene) is a plant-derived aromatic compound that belongs to the stilbenoid family and has a wide range of beneficial properties including anti-inflammatory and anti-oxidative effects (Bo et al., 2013). Moreover, it has been shown to beneficially influence the expression of disease biomarkers of diabetes, cardiovascular diseases, and neurological disorders (Bhatt et al., 2012; Brasnyó et al., 2011; Tomé-Carneiro et al., 2012). Resveratrol and its derivatives are commercialized as components of certain nutritional supplements, healthy foods, and cosmetics. In addition, several recent studies have reported new applications of these compounds as food additives and functional polymer materials (Mora-Pale et al., 2015; Tian et al., 2020; Zhang et al., 2015). Therefore, further growth of the resveratrol market is expected. Resveratrol currently available on the market is predominantly extracted from certain plants including grape, peanut, and the Japanese knotweed *Polygonum cuspidatum* (Chen et al., 2013; Chukwumah et al., 2009; Li et al., 2017). However, due to the unstable harvest of these plants and the low resveratrol purity obtained, it is necessary to develop a stable production process of high-purity resveratrol from inexpensive feedstocks.

**Fig. 1:**
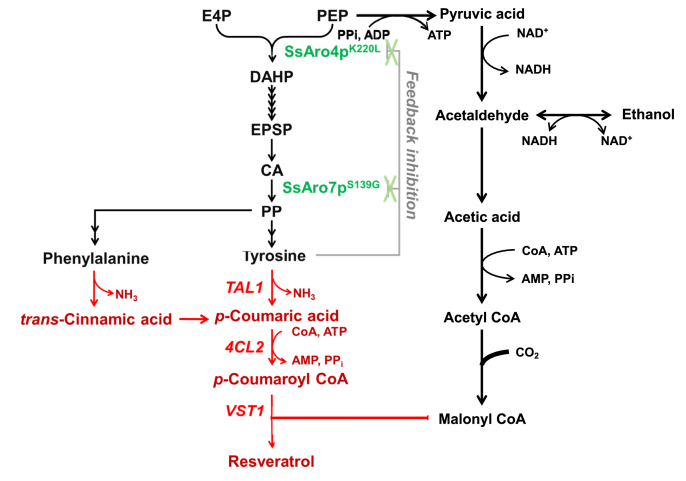
Construction of resveratrol-producing yeast strain using *S. stipitis*. Biosynthetic pathway towards resveratrol in the engineered *S. stipitis*. Native reactions and metabolites are shown in back, the heterologous ones in red, and those that were not used in this study in blue. The mutants of endogenous enzymes overexpressed in the engineered strains are shown in green. E4P: erythrose-4-phosphate; PEP: phosphoenolpyruvate; DHAP: 3-deoxy-D-arabino heptulosonate-7-phosphate; CA: chorismic acid; PP: prephenate. Abbreviation of enzymes, *TAL1*: tyrosine ammonia-lyase; *4CL2*: 4-coumarate: CoA ligase; *VST1*: resveratrol synthase; SsAro4p: DAHP synthase from *S. stipitis*; SsAro7p: chorismate mutase from *S. stipitis*. (For interpretation of the references to color in this figure legend, the reader is referred to the Web version of this article.)

Bioproduction of resveratrol by recombinant microorganisms is a promising approach and a potential solution to the current issues associated with resveratrol production (Braga et al., 2018; Kallscheuer et al., 2016; Li et al., 2016a, Li et al., 2016b; Sáez-Sáez et al., 2020; Wang et al., 2018; Wu et al., 2017). Resveratrol is typically biosynthesized from the aromatic amino acids L-phenylalanine (L-Phe) or L-tyrosine (L-Tyr), both derived from the shikimate pathway (Fig. 1). The first biosynthetic step involves the deamination of phenylalanine or tyrosine, via the phenylalanine/tyrosine ammonia lyase (PAL/TAL), to produce *trans*-cinnamic acid or *p*-coumaric acid, respectively (Sparvoli et al., 1994). The *trans*-cinnamic acid is subsequently hydroxylated to *p*-coumaric acid by cinnamic acid hydroxylase (C4H) and a cytochrome P450 enzyme (Li et al., 2016a, Li et al., 2016b). Then *p*-coumaric acid is attached to coenzyme A (CoA) by 4-Coumarate: CoA ligase (4CL) to form *p*-coumaroyl CoA using ATP (Stuible et al., 2000). Finally, resveratrol synthase (VST) condensates one molecule of *p*-coumaroyl CoA with three molecules of malonyl CoA to synthesize resveratrol (Winkel-Shirley, 2001).

Yeasts such as *Saccharomyces cerevisiae* are an attractive option for the production of valuable plant-derived compounds, including resveratrol, because of their robustness and tolerance to harsh fermentation conditions. Moreover, yeasts can provide a physiological environment suitable for the functional expression of plant-derived heterologous enzymes, as they allow endomembrane localization and post-translational modifications (Hammer and Avalos, 2017; Srinivasan and Smolke, 2020; Xue et al., 2013). Indeed, several studies have reported successful production of resveratrol from glucose using the recombinant yeast species *S. cerevisiae* and the oleaginous yeast *Yarrowia lipolytica* (Li et al., 2016a, Li et al., 2016b; Sáez-Sáez et al., 2020). On the other hand, it has also been reported that the production of noscapine, a plant-derived benzylisoquinoline alkaloid synthesized from L-Tyr, by recombinant *S. cerevisiae* increased over 50-fold when disaccharide trehalose was used as carbon source instead of glucose (Li et al., 2018). This finding suggests that the yield of shikimate pathway-derived aromatic compounds including resveratrol could also be influenced by the carbon sources. Nevertheless, due to the limited substrate utilization capacity of *S. cerevisiae* and *Y. lipolytica*, the effects of different carbon sources on resveratrol production have not been fully investigated.

*Scheffersomyces stipitis* (formerly known as *Pichia stipitis*) is a crabtree-negative yeast that habits the gut of wood-decaying beetles (Nardi et al., 2006). Moreover, *S. stipitis* genome encodes the enzymes and transporters necessary to dissimilate a wide variety of saccharides (Jeffries and Van Vleet, 2009). In fact, this yeast can use a broad range of biomass-derived sugars as carbon sources, including fructose, xylose, *N*-acetyl glucosamine (GlcNAc), mannose, galactose, cellobiose, maltose, and sucrose (Inokuma et al., 2016; Jeffries et al., 2007; Preez et al., 1986); and is a frequently used host for bioethanol production based on the utilization of these sugars (Inokuma et al., 2016; Jeffries, 2014; Nigam, 2001; Slininger et al., 2006). Furthermore, the recombinant yeast constructed based on *S. stipitis* showed significantly higher shikimic acid production capacity than the strain based on *S. cerevisiae* (Gao et al., 2017). These reports showed the high potential of *S. stipitis* for the production of shikimate pathway-derived compounds from various sugars.

Here, we aimed to assess the production of resveratrol from 8 different types of sugars (glucose, xylose, galactose, fructose, GlcNAc, maltose, sucrose, and cellobiose) as carbon source by an engineered *S. stipitis*. Genes encoding essential enzymes for resveratrol biosynthesis were introduced in the yeast and specific feedback-insensitive alleles were overexpressed to improve resveratrol production in the constructed strain. Furthermore, the metabolic responses during fermentation of the different carbon sources were quantitatively investigated by capillary electrophoresis time-of-flight mass spectrometry (CE-TOF-MS).

## 2 Materials and methods

### 2.1 Strains and media

The DH5*α* strain of *Escherichia coli* (Toyobo, Osaka, Japan) was used for plasmids construction and amplification. DH5*α* was routinely cultivated in LB medium (10 g/L tryptone, 5 g/L yeast extract, and 10 g/L NaCl) containing 100 μg/mL ampicillin at 37 °C.

*S. stipitis* NBRC10063 strain was obtained from the NITE Biological Resource Center (NBRC). Selection and pre-cultivation of yeast transformants was conducted in synthetic dextrose (SD) medium [6.7 g/L of yeast nitrogen base without amino acids (Difco Laboratories, Detroit, MI, USA) and 20 g/L of glucose] supplemented with appropriate amino acids and nucleic acids at 30 °C. All fermentations were performed in the YP medium (10 g/L yeast extract; 20 g/L peptone) containing 50 g/L of glucose, xylose, galactose, fructose, GlcNAc, maltose, cellobiose, or sucrose, which were designated YPD50, YPX50, YPGal50, YPF50, YPGN50, YPM50, YPC50, and YPS50, respectively. The genetic properties of yeast strains used in this study are shown in Table 1.

**Table 1:** Yeast strains and plasmids.

Strains and plasmids	Relevant genotype	Ref
*Strains*		
*S. stipitis* NBRC10063	Wild type	NBRC
Ss101	*S. stipitis, ΔURA5*	This study
Ss102	*S. stipitis, ΔURA5, ΔADE2*	This study
Ss-T4V	Ss102{pInA2-T4V}, *ΔURA5*	This study
Ss-T4V-em	Ss102{pInA2-T4V, pInU5-em}	This study
Ss-T4V-aro4m	Ss102{pInA2-T4V, pInU5-ARO4m}	This study
Ss-T4V-aro7m	Ss102{pInA2-T4V, pInU5-ARO7m}	This study
Ss-T4V-aro47m	Ss102{pInA2-T4V, pInU5-ARO47m}	This study
*Plasmids*		
pUC19	*Amp*^*R*^	Lab stock
pCU5-cas9	*Amp*^*R*^*, URA5, ARS1, CEN6, PIR1p-Cas9-GLN1t*	This study
pCU5-cas9-Ade2	*Amp*^*R*^*, URA5, ARS1, CEN6, PIR1p-Cas9-GLN1t, gRNA for ADE2*	This study
pInA2-T4V	*Amp*^*R*^*, ADE2, PIR1p-HaTAL1-TEF1t, ENO1p-At4CL2-UAGt, TEF1p-VvVST1-GLN1t*	This study
pInU5-em	*Amp*^*R*^*, URA5, PIR1p, ENO1t*	This study
pInU5-ARO4m	*Amp*^*R*^*, URA5, PIR1p-SsARO4*^*K220L*^*-TEF1t*	This study
pInU5-ARO7m	*Amp*^*R*^*, URA5, ENO1p-SsARO7*^*G139S*^*-UAGt*	This study
pInU5-ARO47m	*Amp*^*R*^*, URA5, PIR1p-SsARO4*^*K220L*^*-TEF1t, ENO1p-SsARO7*^*G139S*^*-UAGt*	This study

### 2.2 Gene synthesis

*TAL1* derived from *Herpetosiphon aurantiacus* (*HaTAL1*), *4CL2* derived from *Arabidopsis thaliana* (*At4CL2*), *VST1* derived from *Vitis vinifera* (*VvVST1*), and *cas9* genes were codon-optimized for *S. stipitis* and synthesized by GeneArt (Invitrogen, Waltham, MA, USA), respectively.

### 2.3 Plasmid construction

The plasmids used in this study are listed in Table 1. Details on plasmid construction have been provided as Supplementary Text S1. All primers used for construction are listed in Supplementary Table S1.

### 2.4 Construction of auxotrophic and resveratrol producing strains

*S. stipitis* transformation was performed using the lithium-acetate method (Gietz and Woods, 2002) with a minor modification in which the incubation time at 42 °C was reduced from 30 min to 5 min. To construct the uracil auxotrophic strain, the NBRC10063 strain cultured in YPD medium for 24 h was spread on an SD medium plate with 1 mg/L 5-fluoroorotic acid (5-FOA) and 200 mg/L uracil. The colonies grown on this condition were restreaked on SD medium plate containing 20 mg/L uracil. Then, an uracil auxotrophic strain was identified using SD plates with or without 20 mg/L uracil. The frame-shift mutation of *URA5* gene was confirmed by sequencing. The identified strain (*ΔURA5*) was designed as Ss101. To construct an adenine auxotrophic strain based on Ss101, the plasmid for expression of cas9 and guide RNA (gRNA) for the *ADE2* target site pCU5-cas9-ade2 was transfected into the Ss101 strain and screened on SD medium plate containing 100 mg/L adenine hemisulfate. The colonies were restreaked into SD medium plate containing 20 mg/L adenine hemisulfate, and their color was evaluated to confirm whether they get the adenine auxotrophy. The frame-shift mutation of *ADE2* gene was confirmed by sequencing the target genome locus. The resulted strain (*ΔURA5*/*ΔADE2*) was designated as Ss102.

For the construction of the resveratrol producing strain, pIA2-T4V plasmid was linearized with *PshA*Ⅰ, transfected into the Ss102 strain, and integration in the *ADE2* locus of its genomic DNA by single crossover recombination, yielding the Ss-T4V strain. Then, pIU5-em, pIU5-ARO4m, pIU5-ARO7m, and pIU5-ARO47m were linearized with *BtgZ*Ⅰ, transfected into the Ss-T4V strain, and integrated in the *URA5* locus of its genomic DNA by single crossover recombination to produce the Ss-T4V-em, Ss-T4V-aro4m, Ss-T4V-aro7m, and Ss-T4V-aro47m strains, respectively.

### 2.5 Quantitative real-time PCR (qRT-PCR)

Yeast harvested from the certain cultures were used for RNA preparation to observe the gene expression. Total RNA was extracted from the cells harvested at 48 h fermentation using the NucreoSpin RNA (Macherey–Nagel, Düren, Germany) according to the manufacturer’s instructions. The expression levels of *ARO4* and *ARO7* genes were quantified by real-time PCR as previously described (Inokuma et al., 2018). All the primers used for qRT-PCR are listed in Supplementary Table S1.

### 2.6 Fermentation methods

Fermentations for resveratrol production by the engineered strains were performed in 200 mL baffled flasks containing 30 mL of certain medium at 30 °C in an orbital shaker incubator (100 rpm; BR-43FL; Taitec, Saitama, Japan). Pre-cultivated yeast cells were inoculated at an initial OD_600_ of 0.1. The culture broth was sampled every 24 h, and the OD_600_, and concentrations of sugars, ethanol, resveratrol, and *p*-coumaric acid were measured. For resveratrol and *p*-coumaric acid extraction, equal volumes of absolute ethanol and culture medium were mixed, and centrifuged at 13,000 rpm for 5 min (Li et al., 2016a, Li et al., 2016b). The supernatants were used to analyze resveratrol and *p*-coumaric acid concentration.

### 2.7 Analytic methods

Glucose, xylose, fructose, galactose, GlcNAc, and ethanol concentrations in the culture supernatant were analyzed with an HPLC (Shimadzu, Kyoto, Japan) equipment with a Aminex HPX-87H column (7.8mm × 300 mm, particle size 9 μm; Bio-Rad, Hercules, CA, USA) and an RID-10A refractive index detector (Shimadzu). The HPLC system was operated at 65 °C, with 5 mM H_2_SO_4_ as the mobile phase at a flow rate of 0.6 mL/min.

Cellobiose, maltose, and sucrose concentrations analyzed by an HPLC system (Shimadzu) equipped with a Concil Sugar-D column (4.6mm × 250 mm, particle size 5 μm; Nacalai tesque Inc., Kyoto, Japan) and an RID-10A refractive index detector (Shimadzu). The HPLC system was operated at 30 °C, with 70% acetonitrile as the mobile phase at a flow rate of 1 mL/min.

The concentration of resveratrol and *p*-coumaric acid extracts were analyzed with an HPLC (Shimadzu, Kyoto, Japan) equipment with a Luna Omega PS C18 (150 × 4.6 mm, particle size 3 μm Phenomenex, CA, USA) and an SPD-20A UV/VIS detector (Shimadzu). The HPLC system was operated at 30 °C, with the solvent A (0.1% formic acid) and the solvent B (acetonitrile) at a flow rate of 1 mL/min. Linear gradient from 0% to 90% of solvent A over 0.5–9.5 min was used. Resveratrol and *p*-coumaric acid were detected by absorbance at 304 nm. Dry cell weight (DCW; g/L) of *S. stipitis* strains was calculated by multiplying the values of OD_600_ by 0.300 according to the constructed calibration curve (Supplementary Fig. S1).

### 2.8 CE-TOF-MS analysis

After 48 h fermentation, 2 mL of the cultures were obtained and the intracellular metabolites were extracted according to a previously reported method (Inokuma et al., 2018). The extracts were dried in a vacuum evaporator (CVE-3100, Tokyo Rikakikai, Osaka, Japan) overnight and stored at −80 °C until use. The dried metabolites were dissolved in ultrapure water and the concentrations of the anionic and cationic intermediates were measured by CE-TOF-MS as previously described (Hasunuma et al., 2013).

### 2.9 α-Glucosidase activity assay

After 48 h fermentation in 30 mL of YPM50 medium, yeast cells were separated from the culture supernatant by centrifugation at 1000×*g* for 10 min, washed twice with distilled water, and centrifuged again at 1000×*g* for 5 min. The washed cells were treated with a cell-wall degrading enzyme (lyticase, CelLytic Y Plus kit, Sigma–Aldrich, St. Louis, MO, USA) to extract cell wall proteins and spheroplasts were removed by centrifugation as described previously (Bamba et al., 2018). The supernatant was used as the cell wall protein sample. The pellet containing spheroplasts were broken using Shake Master Neo (Bio Medical Science, Tokyo, Japan) and 0.6 mm glass beads to prepare cell extracts, followed by centrifugation at 9300×*g* for 5 min to remove the spheroplast lysates. The supernatant was used as the intracellular protein sample.

α-Glucosidase activity of the culture supernatant, the cell wall protein, and the intracellular protein samples were evaluated by using Glucose Forming Activity Assay Kit (Kikkoman Corp., Chiba, Japan), as described previously (Yamakawa et al., 2012). One unit of glucoamylase activity was defined as the amount of enzyme required to liberate 1 μmol of *p*-nitrophenol per minute. The enzyme activity was normalized by the original volume of the culture medium.

### 2.10 Statistical analyses

The data are presented as the mean of three independent replicates. For assessment significant differences between culture conditions (Fig. 5), the one-way ANOVA was used as a statistical test in conjunction with Tukey's range test. The statistical analysis was performed in EZR (Kanda, 2013) which is a modified version of R commander designed to add statistical functions frequently used in biostatistics. A *p* < 0.05 was considered statistically significant. A principal component analysis (PCA) was conducted using MetaboAnalyst (https://www.metaboanalyst.ca).

## 3 Results

### 3.1 Construction of the recombinant *S. stipitis* strain for resveratrol production

**Fig. 2:**
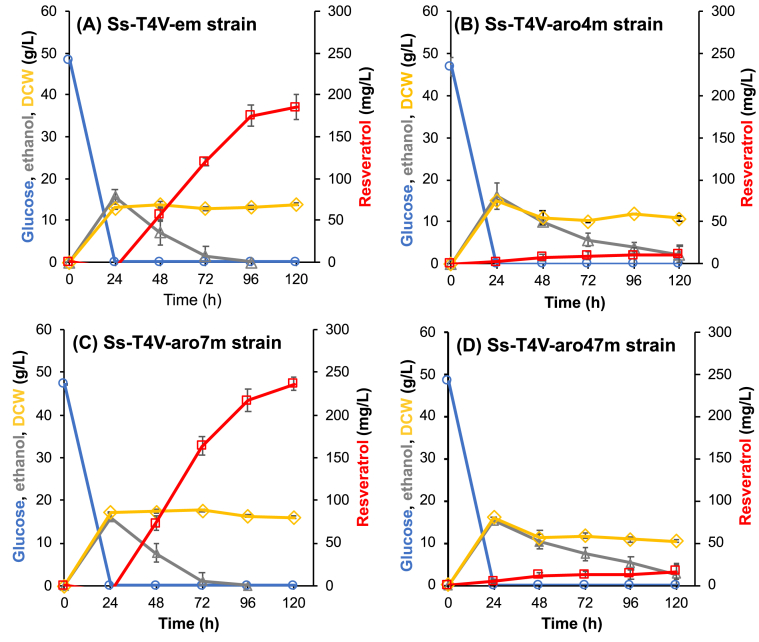
Time course of microbial production of resveratrol by the engineered strains. Fermentation was performed in YP medium with 50 g/L of glucose by (A) the control strain expressing *HaTAL1*, *At4CL2* and *VvVST1*; (B) Ss-T4V-aro4m strain overexpressing *SsARO4*^*K220L*^; (C) Ss-T4V-aro7m strain overexpressing *SsARO7*^*G139S*^; and (D) Ss-T4V-aro47m strain overexpressing both *SsARO4*^*K220L*^ and *SsARO7*^*G139S*^. The values and error bars represent mean ± standard deviation of three independent experiments.

For the construction of the resveratrol producing strain using *S. stipitis* as a host, the expression cassettes of tyrosine ammonia-lyase gene from *H. aurantiacus* (*HaTAL1),* 4-coumarate: CoA ligase gene from *A. thaliana* (*At4CL2*), and stilbene synthase gene from *V. vinifera* (*VvVST1*) were first constructed under the control of the endogenous promoters: *PIR1p*, *ENO1p*, and *TEF1p*, respectively and these expression cassettes were integrated into the *ADE2* locus of the genome of the adenine and uracil auxotrophic *S. stipitis* strain (Ss102) to construct Ss-T4V strain. After integrating the empty vector (pIU5-em) into the *URA5* locus of the genome of this strain, the fermentation test with the resulting strain (Ss-T4V-em) was performed with 200 mL baffled flask containing 30 mL of YPD50 medium. The Ss-T4V-em strain consumed all 50 g/L of glucose, and 18.0 g/L of ethanol were detected at 24 h. After glucose exhaustion, consumption of the produced ethanol and production of resveratrol were observed, this resulted in a titer of approximately 174.8 mg/L resveratrol after 120 h of fermentation (Fig. 2A).

### 3.2 Improvement of the flux of the shikimate pathway for resveratrol production

Genes encoding 3-deoxy-D-arabino-heptulosonate-7-phosphate (DAHP) synthase (*ARO4*) and chorismate mutase (*ARO7*) are common engineered targets of shikimate pathway-derived products, including resveratrol (Li et al., 2015; Sáez-Sáez et al., 2020; Suástegui and Shao, 2016). In order to improve the metabolic flux toward the shikimate pathway, a gene encoding feedback-inhibition insensitive variant of DAHP synthase from *S. stipitis* (*SsARO4*^*K220L*^) (Gao et al., 2017) was overexpressed in the Ss-T4V strain, resulting in the construction of the Ss-T4V-aro4m strain. The overexpression of *SsARO4*^*K220L*^ was confirmed by qRT-PCR (Supplementary Fig. S2). Contrary to the results of a previous study on shikimic acid production by recombinant *S. stipitis* carrying the feedback-inhibition insensitive DAHP synthase alleles *SsARO4*^*K220L*^ (Gao et al., 2017); in our study, *SsARO4*^*K220L*^ overexpression resulted in an significantly decrease of resveratrol production (10.3 mg/L) and in growth inhibition of *S. stiptis* (Fig. 2B).

We also constructed a strain expressing the gene encoding a feedback-inhibition insensitive variant of chorismate mutase from *S. stipitis* (*SsARO7*). Since there are no reports on the feedback-inhibition insensitive variant of *SsARO7*, firstly, the amino acid sequence of this protein was aligned with the chorismate mutase from several yeasts and bacteria, and the target mutational site of *SsARO7* for resistance of feedback inhibition (G139) was identified (Supplementary Fig. S3). Based on the analysis, we constructed a novel feedback-inhibition insensitive variant (*SsARO7*^*G139S*^) and overexpressed it in the Ss-T4V and Ss-T4V-aro4m strains, resulting in the construction of Ss-T4V-aro7m and Ss-T4V-aro47m strains, respectively. The overexpression of *SsARO7*^*G139S*^ in these strains was confirmed by qRT-PCR (Supplementary Fig. S2). Ss-T4V-aro7m and Ss-T4V-aro47m produced approximately 236.5 mg/L (Figs. 2C) and 16.4 mg/L (Fig. 2D) of resveratrol, respectively. Based on this result, Ss-T4V-aro7m strain, which showed the highest resveratrol titer, was used for the following experiments of resveratrol production from biomass-derived sugars.

### 3.3 Resveratrol production from biomass-derived mono-/disaccharides

**Fig. 3:**
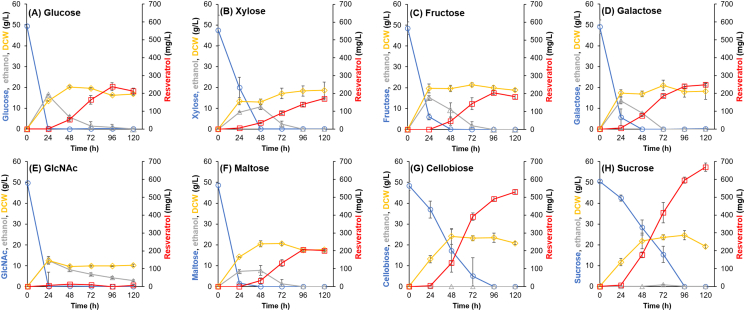
Resveratrol production from mono-/disaccharides. Time course of the fermentations performed by the Ss-T4V-aro7 strain on YP medium with 50 g/L of (A) glucose, (B) xylose, (C) fructose, (D) galactose, (E) GlcNAc, (F) maltose, (G) cellobiose, and (H) sucrose.

To verify the potential of resveratrol production from biomass-derived mono-/disaccharides using the recombinant *S. stipitis* strain, fermentation with the Ss-T4V-aro7m strain was conducted in the YP media containing 50 g/L of glucose, fructose, galactose, xylose, GlcNAc, cellobiose, maltose, or sucrose as carbon source. The Ss-T4V-aro7m strain successfully produced resveratrol from all the sugars tested. During glucose, fructose, galactose, xylose, and maltose fermentation 50 g/L of these sugars were almost completely consumed, and 7.2–18.0 g/L of ethanol was detected after 24 h (Fig. 3A, B, C, D, and F). In addition, in the early phase of maltose fermentation, a transient accumulation of glucose derived from maltose was detected in the culture supernatant (Supplementary Fig. S4). Resveratrol production reached titers of 237.6, 204.6, 170.6, 248.6, and 206.9 mg/L, with overall yields of 4.8, 4.2, 5.1, 3.6, and 4.3 mg/g carbon source, in the medium containing glucose, fructose, galactose, xylose, or maltose, respectively at 120 h (Fig. 3). Moreover, ethanol concentration decreased after 24 h. On the other hand, during cellobiose and sucrose fermentation, the 50 g/L of these sugars were gradually consumed for 96 h and no ethanol production was observed. For cellobiose and sucrose fermentation, the final titers and overall yields of resveratrol were 529.8 and 668.6 mg/L, and 11.0 and 13.2 mg/g carbon source, respectively (Fig. 3G and H). No significant accumulation of monosaccharides derived from cellobiose and sucrose hydrolysis (glucose and fructose) was observed in the culture supernatants (Supplementary Fig. S4).

Among the sugars tested in this study, GlcNAc resulted in the lowest resveratrol titer (13.78 mg/L) at 48 h fermentation, while showing remarkable accumulation of *p*-coumaric acid (116.0 mg/L) after 120 h (Fig. 3E and Supplementary Fig. S5).

### 3.4 Intracellular metabolomic analysis under disaccharides dissimilation condition

**Fig. 4:**
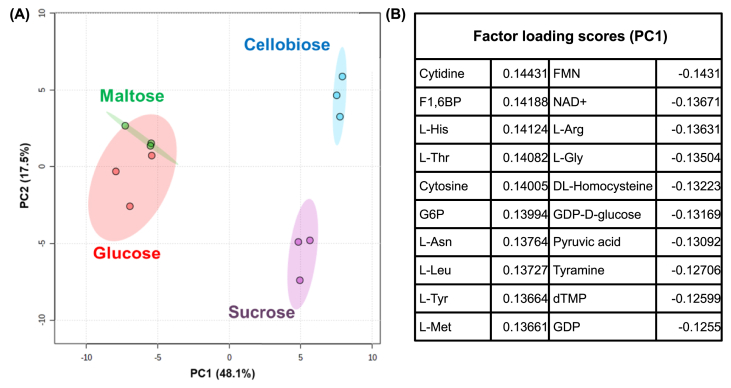
Principal component analysis (PCA) of the metabolomic data of Ss-T4V-aro7m strain after 48 h fermentation with four different carbon sources (glucose, cellobiose, maltose, or sucrose). (A) PCA score plot, (B) The top 20 metabolites with highest factor loading scores corresponding to PC1.

To further investigate the metabolic response on the improved resveratrol productivity using cellobiose and sucrose as carbon sources, the intracellular metabolites of the Ss-T4V-aro7m strain were extracted after 48 h of fermentation in YPD50, YPC50, YPM50, and YPS50 media, and metabolomic analysis was conducted using CE-TOF-MS. A total of 96 metabolites involved in glycolysis, pentose phosphate pathway, TCA cycle, nucleic and amino acid syntheses, and in energy and cofactor metabolisms, were detected in at least one of the fermentation tests listed in Supplementary Table S3. Using these data, we firstly performed a principal component analysis (PCA) to obtain an overview of the metabolic profile (Fig. 4A). Factor loadings corresponding to principal component 1 (PC1) and 2 (PC2) are summarized in Supplementary Table S3. PC1 of the PCA accounted for 48.1% of the variability and the PC1 axis separated the data cluster of the glucose/maltose and cellobiose/sucrose fermentation. Among the metabolites with high contributions to PC1, flavin mononucleotide (FMN), NAD^+^, L-arginine, L-glycine, DL-homocysteine, guanosine diphosphate (GDP), pyruvic acid, tyramine, thymidine monophosphate (dTMP), and GDP-D-glucose were detected at higher levels during glucose and maltose fermentation, whereas cytidine, fructose-1,6-bisphosphate (F1,6BP), L-threonine, L-histidine, cytosine, glucose-6-phosphate (G6P), L-asparagine, L-leucine, L-Tyr, and L-methionine showed higher levels of metabolite on during cellobiose and sucrose fermentation (Fig. 4B).

**Fig. 5:**
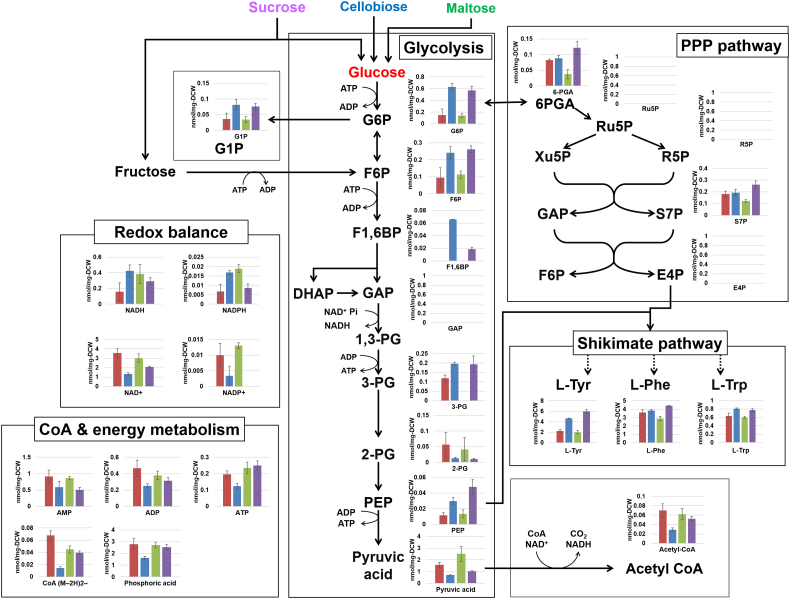
Concentration (nmol/mg DCW) of metabolites involved in the glycolytic pathway and central metabolism of Ss-T4V-aro7m strain after 48 h of glucose (red bars), cellobiose (blue bars), maltose (green bars) or sucrose (purple bars) fermentation. The arrows indicate the main direction of metabolic flow. Dashed arrows indicate reactions involving more than one enzymatic step. Data are presented as means ± standard deviation (n = 3). G1P: glucose-1-phosphate; G6P: glucose-6-phosphate; F6P: fructose-6-phosphate; F1,6BP: fructose-1, 6-bis-phosphate; DHAP: dihydroxyacetone phosphate; GAP: glyceraldehyde 3-phosphate; 1,3-PG: 1,3-bisphosphoglycerate; 2-PG: 2-phosphoglycerate; 3-PG: 3-phosphoglycerate; PEP: phosphoenolpyruvate; 6-PGA: 6-phosphogluconate; E4P: erythrose-4-phosphate; Ru5P: ribulose-5-phosphate; R5P: ribose-5-phosphate; S7P: sedoheptulose-7-phosphate; Xu5P: xylulose-5-phosphate; NAD^+^: nicotinamide adenine dinucleotide; NADP^+^: nicotinamide adenine dinucleotide phosphate. (For interpretation of the references to color in this figure legend, the reader is referred to the Web version of this article.)

The states of central metabolism of the Ss-T4V-aro7m strain were summarized in the metabolites map (Fig. 5). Elevated accumulation of glycolytic metabolites G6P, F6P, F1,6BP, 3-PG, and PEP was detected on the fermentation with cellobiose and sucrose, while high amounts of pyruvic acid were accumulated during glucose and maltose fermentation. Moreover, the levels of aromatic amino acids synthesized via the shikimate pathway, especially L-Tyr, the intermediate for resveratrol production, were increased on the fermentation with cellobiose and sucrose. During cellobiose and sucrose fermentations, the levels of NAD^+^ and NADP^+^ were lower than those detected in maltose and glucose fermentations (Fig. 5). This suggested an altered redox balance in *S. stipitis* under fermentation with different sugars.

## 4 Discussion

Yeasts are recognized as promising host microorganisms for chemicals and biofuels production from biomass feedstocks (Do et al., 2019; Yaguchi et al., 2018; Zha et al., 2021) and many research groups have successfully produced resveratrol from glucose using yeast species such as *S. cerevisiae* and *Y. lipolytica* as a host for construction of the recombinant resveratrol producing strains (Li et al., 2016a, Li et al., 2016b; Sáez-Sáez et al., 2020)*.* However, there have been no reports on the use of biomass-derived sugars, other than glucose, as carbon sources for resveratrol production, and the effects of the assimilation of these sugars on resveratrol production have not been investigated. In this study, we employed a Crabtree-negative yeast *S. stipitis*, which has a broad range of biomass-derived sugars utilization capacity, to construct recombinant strains for resveratrol production and successfully produced resveratrol from a broad range of sugars (glucose, fructose, galactose, xylose, GlcNAc, cellobiose, maltose, and sucrose). To the best of our knowledge, this is the first study assessing resveratrol production from sugars different from glucose.

We also found that resveratrol production by the recombinant yeast greatly varied depending on the sugar used as substrate (Fig. 3). In particular, resveratrol titers when cellobiose and sucrose were used as substrates (529.8 and 668.6 mg/L, respectively) were more than two times higher than those obtained from glucose (237.6 mg/L). On the other hand, in maltose fermentation, which is also a disaccharide, the resveratrol titer was similar or lower to those of the monosaccharides tested, including glucose. These results indicate that the cellular response of the engineered *S. stipitis* during cellobiose and sucrose fermentation were significantly different to those observed with the other monosaccharides and maltose. When monosaccharides and maltose were used as the carbon sources, the engineered *S. stipitis* immediately consumed almost all the sugar present in the medium (24 h) and produced ethanol at relatively high yields (27–62% of the theoretical maximum yield). After saccharides depletion, this strain consumed the produced ethanol, and simultaneously produced the resveratrol. This result is consistent with the previous study on the resveratrol production from glucose by the recombinant *S. cerevisiae* (Li et al., 2015). Moreover, in maltose fermentation, transient glucose accumulation in the supernatant was observed (Supplementary Fig. S4), suggesting that maltose was partially hydrolyzed to glucose extracellularly by a hydrolytic enzyme (α-glucosidase) and transported into the yeast as glucose. We measured the α-glucosidase activity of the culture supernatant, the cell wall protein, and the intracellular protein samples prepared from the maltose fermentation with the Ss-T4V-aro7m strain (Supplementary Fig. S6). We detected the α-glucosidase activity, most of which was extracellularly (culture supernatant and cell wall). This result strongly supports extracellular hydrolysis of maltose to glucose in *S. stipitis*. On the other hand, during cellobiose and sucrose fermentation, the engineered *S. stipitis* showed moderate sugar consumption rates and no ethanol and monosaccharide accumulation in the supernatant was observed (Fig. 3 and Supplementary Fig. S4). This clearly indicated that the prevention of catabolite repression along with slow consumption of substrates would enhance resveratrol titers.

To further understand the cellular response during disaccharides fermentations, the metabolites produced during the fermentations of maltose, cellobiose, sucrose, and glucose were analyzed. We found different patterns of accumulation of metabolites involved in the glycolysis pathway. In particular, the accumulation of PEP during cellobiose and sucrose fermentations were higher than those observed in maltose and glucose fermentations. In contrast, pyruvate was significantly accumulated under glucose and maltose fermentations (Fig. 5). This suggests that conversion of PEP to pyruvic acid by pyruvate kinase (PYK) was strongly inhibited under cellobiose and sucrose fermentations to prevent that the overflow of pyruvic acid exceed the respiratory capacity. PYK is an important enzyme and its activity is strictly regulated by phosphorylation and allostery in various organisms such as yeast and mammals (El-Maghrabi et al., 1982; Galello et al., 2010; Jurica et al., 1998). A previous study on PYK regulation in *S. cerevisiae* demonstrated that glucose depletion causes the decrease of PYK activity and accumulation of PEP (Xu et al., 2012). In cellobiose and sucrose fermentations, where there was no accumulation of monosaccharides in the supernatant (Supplementary Fig. S4), PYK activity was strictly regulated, and probably more PEP was entering the shikimic acid pathway. The elevated levels of aromatic amino acids synthesized via the shikimate pathway under cellobiose and sucrose culture conditions also support this hypothesis.

The Ss-T4V-aro7m strain produced more than 100 mg/L of resveratrol from most of the sugars tested in this study, except from the amino sugar GlcNAc (13.78 mg/L at 48h, Fig. 3E). On the other hand, this strain produced *p*-coumaric acid from GlcNAc at a concentration of 116 mg/L (0.71 mM) (Supplementary Fig. S3), which is comparable to the molar concentration of resveratrol produced from xylose (0.75 mM). These results suggest that the reaction downstream *p*-coumaric acid is a bottleneck step in resveratrol production under GlcNAc assimilation condition. In the Ss-T4V-aro7m strain, *At4CL2* and *VvVST1* were expressed under the control of the endogenous *ENO1p* and *TEF1p*, respectively. These promoters have been reported to be expressed constitutively under glucose and xylose assimilation conditions (Gao et al., 2017), while their expression levels under assimilation conditions of other sugars, including GlcNAc, have not been investigated. Investigation of the expression levels of various promoters under the GlcNAc assimilation condition and balanced expression of genes involved in the resveratrol biosynthetic pathway may improve resveratrol productivity from GlcNAc. Another possible cause of the low resveratrol production is the elevated oxidative stress in the GlcNAc assimilation condition as it has been reported that GlcNAc-grown *S. stipitis* cells are exposed to higher levels of oxidative stress than glucose-grown cells (Inokuma et al., 2018). Additionally, Braga et al. (2018) have previously reported that high oxidative stress could cause oxidation or multimerization of resveratrol. Therefore, the resveratrol production from GlcNAc by *S. stipitis* may also be increased by reducing oxidative stress.

In the present study, we also constructed a novel feedback-inhibition insensitive variant of chorismate mutase from *S. stipitis* (SsAro7p^G139S^). Overexpression of SsAro7p^G139S^ successfully contributed to enhance resveratrol production in recombinant *S. stipites*, resulting in a 1.3-fold increase of resveratrol production compared with the parental strain, and titers of 236.5 mg/L resveratrol from glucose (Fig. 2). On the other hand, despite a previous study reported a successful improvement of shikimic acid production by introducing *SsARO4*^*K220L*^ in *S. stipitis* (Gao et al., 2017), in our study the introduction of *SsARO4*^*K220L*^ in the constructed strain significantly decrease resveratrol production and cause growth inhibition. We hypothesized that in resveratrol production by the recombinant *S. stipitis*, PEP metabolic pool is strictly limited and thus overexpression of *ARO4*^*K220L*^ leads to lack of PEP for cell growth and malonyl CoA supply. Further studies to optimize the expression level of *SsARO4*^*K220L*^ may allow us to avoid growth inhibition and improve resveratrol productivity in *S. stipitis*.

Literatures on resveratrol production in recombinant yeasts from sugar substrates are summarized in Table 2. The production of resveratrol from cellobiose and sucrose by Ss-T4V-aro7m in this study (529.8 and 668.6 mg/L, respectively) was higher than that from glucose in batch fermentation using recombinant *S. cerevisiae* and *Y. lipolytica* (272.64 and 524.9 mg/L, respectively) reported previously. These studies in *S. cerevisiae* and *Y. lipolytica* have conducted wider metabolic engineering such as increasing copy number of the resveratrol pathway genes and overexpressing acetyl CoA carboxylase to increase malonyl CoA supply, whereas Ss-T4V-aro7m achieved higher production with only introducing a single copy of the resveratrol biosynthetic pathway and a feedback-insensitive *ARO7* mutant. This shows the high potential of *S. stipitis* as a microbial platform for resveratrol production and also suggests that additional metabolic engineering can further increase the resveratrol production level of this yeast. On the other hand, the recombinant *S. cerevisiae* and *Y. lipolytica* have improved their resveratrol titer to 812 mg/L and 12.4 g/L, respectively, by feeding glucose (Table 2). Employing fed-batch fermentation and optimization of the fermentation conditions would also be required for the practical application of the resveratrol bio-production process using recombinant *S. stipitis*.

**Table 2:** Resveratrol production in the recombinant yeasts. Table shows different recombinant yeasts for the resveratrol production, including their genetic modifications, substrates uesd, and titers/yields. *SeACS*: acetyl-CoA synthase from *Salmonella enterica*; *AtATR2*: cytochrome P450 reductase from *A. thaliana*; *ScARO4*: DAHP synthase from *S. cerevisiae*; *ScARO7*: chorismate mutase from *S. cerevisiae*; *ScCYB5*: cytochrome *b*5 from *S. cerevisiae*; *ScACC1*: acetyl-CoA carboxylase 1 from *S. cerevisiae*; *ScARO10*: transaminated amino acid decarboxylase from *S. cerevisiae*; *FjTAL*: tyrosine ammonia-lyase derived from *Flavobacterium johnsoniae*; *YlARO4*: DAHP synthase from *Y. lipolytica*; *YlARO7*: chorismate mutase from *Y. lipolytica.*

Yeast strain	Genetic modifications	*Substrate*/*cultivation*	*Titer*/*yield*	Reference
*S. cerevisiae* ST4990	2 copies integration of (*AtPAL2*, *At4CL2*, *AtC4H*, and *VvVST1*) *SeACS*^*L641P*^ *AtATR2* Overexpression of *ScARO4*^*K229L*^, *ScARO7*^*G141S*^, *ScCYB5*, and *ScACC1*^*S659A, S1157A*^ *ΔARO10*	Glucose /batch culture in shake flask	Titer: 272.64 mg/L Yield: 13.6 mg/g glucose	Li et al., 2016a, Li et al., 2016b
		Glucose /fed-batch culture in bioreactor	Titer: 812 mg/L Yield: 8.87 mg/g glucose	Li et al., 2016a, Li et al., 2016b
*Y. lipolytica* ST9671	5 copies integration of (*FjTAL*, *At4CL1*, and *VvVST1*) Overexpression of *YlARO4*^*K221L*^ and *YlARO7*^*G139S*^	Glucose /batch culture in shake flask	Titer: 524.9 mg/L Yield: 20 mg/g glucose	Sáez-Sáez et al. (2020)
		Glucose /fed-batch culture in bioreactor	Titer: 12.4 g/L Yield: 54.4 mg/g glucose	Sáez-Sáez et al. (2020)
*S. stipitis* Ss-T4V-aro7m	Single copy integration of (*HaTAL1, At4CL2, and VvVST1*) Overexpression of *SsARO7*^*G139S*^	Glucose /batch culture in shake flask	Titer: 238 mg/L Yield: 4.25 mg/g glucose	This study
		Cellobiose /batch culture in shake flask	Titer: 530 mg/L Yield: 10.6 mg/g cellobiose	This study
		Sucrose /batch culture in shake flask	Titer: 669 mg/L Yield: 13.4 mg/g sucrose	This study

In conclusion, as the resveratrol market is expected to further grow, it has become necessary to develop a stable resveratrol production process from inexpensive biomass feedstocks. In this study, we constructed a resveratrol-producing strain using *S. stipitis* as the host (Ss-T4V-aro7m strain) and successfully produced resveratrol from eight different types of sugars. Resveratrol production by the Ss-T4V-aro7m strain greatly varied depending on the type of sugars used as substrate. In this sense, among eight different type of sugars tested in this study, the highest resveratrol titer (668.6 mg/L) and yield (13.2 mg/g carbon source) were achieved when sucrose was used as substrate. Although further genetic engineering and optimization of fermentation conditions are needed to improve resveratrol yield from each sugar, these results of this study indicate that *S. stipitis* are an attractive microbial platform for resveratrol production from a broad range of biomass-derived sugars. Furthermore, metabolomics analysis revealed diverse metabolic responses to different disaccharides (cellobiose, sucrose, and maltose). The metabolomics data gathered in this study will be useful for designing effective genetic engineering strategies to develop novel *S. stipitis* strains for the production of other valuable aromatic compounds. One the other hand, during the fermentation process, yeasts encounter a variety of harmful compounds (such as weak organic acids, furan derivatives, and phenolics) generated during biomass pretreatment. These compounds can inhibit the cell growth, xylose consumption, and ethanol yield of *S. stipitis* (Zhu et al., 2019). For industrial application, it will be necessary to enhance the resistance of *S. stipitis* to these compounds.

## Declaration of interest

The authors declare that they have no competing interests with the contents of this article.

## Author statement

**Yuma Kobayashi:** Writing - Original Draft, Conceptualization, Methodology, Investigation, Visualization. **Kentaro Inokuma:** Writing - Review & Editing, Funding acquisition, Validation, Project administration. **Mami Matsuda:** Investigation. **Akihiko Kondo:** Supervision. **Tomohisa Hasunuma:** Project administration, Funding acquisition, Supervision.

## Declaration of competing interest

The authors declare that they have no competing interests with the contents of this article.
