# Supplementary material for: Resveratrol production from several types of saccharide sources by a recombinant Scheffersomyces stipitis strain
Source: Metab Eng Commun. 2021 Nov 26;13:e00188. doi: 10.1016/j.mec.2021.e00188 (PMC8637140; doi:10.1016/j.mec.2021.e00188)
Supplement: Multimedia component 3 [file mmc3.docx]

**Supplementary materials**

**Resveratrol production from several types of saccharide sources by a recombinant *Scheffersomyces stipitis* strain**

Yuma Kobayashi^a^, Kentaro Inokuma^a^, Mami Matsuda^a^, Akihiko Kondo^a,b,c^, Tomohisa Hasunuma^a,b,^*

^a^ Graduate School of Science, Technology and Innovation, Kobe University, 1-1 Rokkodai-cho, Nada-ku, Kobe 657-8501, Japan

^b^ Engineering Biology Research Center, Kobe University, 1-1 Rokkodai-cho, Nada-ku, Kobe 657-8501, Japan

^c^ Biomass Engineering Program, RIKEN, 1-7-22 Suehiro-cho, Tsurumi-ku, Yokohama, Kanagawa 230-0045, Japan

* Corresponding author:

Tomohisa Hasunuma

Telephone: +81-78-803-6356, Fax: +81-78-803-6362

E-mail: hasunuma@port.kobe-u.ac.jp

**Text S1** Plasmid construction

The plasmids and primers used in this study are listed in Table 1 and Supplementary Table S1, respectively. The centromeric plasmid carrying uracil auxotrophic gene marker was firstly constructed as follows: DNA fragments containing autonomously replicating sequence 2 (*ARS2*) (Yang et al., 1994), centoromeric core sequence of chromosome 6 (*CEN6*) (Cao et al., 2017), and orotate phosphoribosyl transferase gene (*URA5*) were amplified from *S. stipitis* NBRC 10063 genomic DNA by polymerase chain reaction (PCR) using the SsARS2-F/SsARS-R, SsCEN500-F/SsCEN500-R, and SsURA5-F/SsURA5-R primers, respectively. Subsequently, these fragments were assembled with the vector fragment amplified from pUC19 by PCR using Ec-element-F/ Ec-element-R primers by the In-Fusion method. The resulting plasmid was designated as pCU5.

The cas9 and guide RNA (gRNA) expression plasmid was constructed as follows: The specific target site in the phosphoribosylamidoimidazole-succinocarboxamide synthase gene (*ADE2*) was identified using the online tool CRISPRdirect (<https://crispr.dbcls.jp/>). the DNA fragment containing *S. stipitis* codon-optimized cas9 gene was synthesized by GeneArt and amplified by PCR using cas9_opt_stipitis-F/ cas9_opt_stipitis-R primers. Subsequently, the *PIR1* promoter and *GLN1* terminator regions were amplified from *S. stipitis* NBRC 10063 genomic DNA by PCR using PIRp-F/R and GLN1t-F/R primers, respectively. These fragments were joined by standard overlap PCR and then subcloned into the vector fragment amplified from pCU5 by PCR using Int-cas9_pCU5-F/R primers by the In-Fusion method. The resulting plasmid is designated by pCU5-cas9. Next, the DNA fragment containing gRNA sequence for the *ADE2* target site and *SUP4* terminator was synthesized by GeneArt and amplified by PCR using gRNA_ade2_SUP4ter-F/SUP4ter-R primers. The *SNR52* promoter fragment was amplified from *S. stipitis* NBRC 10063 genomic DNA by PCR using IF-SNR52p-F/R primers. These fragments were joined by standard overlap PCR. The gRNA expression cassette (*SNR52p-gRNA-SUP4t*) was then subcloned into the vector fragment amplified from pCU5-cas9 by PCR using Inv-pCU5-cas_gRNA-F/R primers by the In-Fusion method. The resulting plasmid is designated by pCU5-cas9-ade2.

The integrative plasmid carrying adenine auxotrophic gene marker was constructed as follows: The DNA fragment containing *ADE2* was amplified from *S. stipitis* NBRC 10063 genomic DNA by PCR using Int-Ade2_pCU5-F/R primers. Inverse PCR with the Inv-pCU5-F/R primers was performed to replace *ARS2*, *CEN6*, and *URA5* of pCU5 to *ADE2*. These fragments were ligated by the In-Fusion method. The resulting plasmid is designated by pInA2.

The integrative plasmid for expression of the resveratrol biosynthesis pathway was constructed as follows: *S. stipitis* codon-optimized *HaTAL1*, *At4CL2*, and *VvVST1* genes were synthesized by GeneArt. *HaTAL1* was amplified by PCR using Overlap-TAL1-F/R primers. Subsequently, the *PIR1* promoter and *TEF1* terminator regions were amplified from *S. stipitis* NBRC 10063 genomic DNA by PCR using PIRp-F/R and TEF1t-F/R primers, respectively. These fragments were joined by standard overlap PCR to obtain the *HaTAL1* expression cassette (*PIR1p*-*HaTAL1*-*TEF1t*) and then subcloned into the vector fragment amplified from pInA2 by PCR using Inv-pInA2-TAL-F/R primers by the In-Fusion method. The resulting plasmid is designated by pInA2-TAL. Next, *At4CL2* was also amplified by PCR using Overlap-4CL2-F/R primers. Subsequently, the *ENO1* promoter and *UAG* terminator regions were amplified from *S. stipitis* NBRC 10063 genomic DNA by PCR using ENO1p-F/R and UAGt-F/R primers, respectively. These fragments were joined by standard overlap PCR to obtain the *At4CL2* expression cassette (*ENO1p*-*At4CL2*-*UAGt*). Similarly, *VvVST1* was amplified by PCR using Overlap-VST1-F/R primers. Subsequently, the *TEF1* promoter and *GLN1* terminator regions were amplified from *S. stipitis* NBRC 10063 genomic DNA by PCR using TEF1p-F/R and GLN1t-F/R primers, respectively. These fragments were joined by standard overlap PCR to obtain the *VvVST1* expression cassette (*TEF1p*-*VvVST1*-*GLN1t*). These expression cassettes were subcloned into the vector fragment amplified from pInA2-TAL by PCR using Inv-pInA2-TAL_pInA2-T4V-F/R primers by the In-Fusion method. The resulting plasmid is designated by pInA2-T4V.

To construct the overexpression cassette of *Ssaro4_K220L_* gene, DNA fragments of *Ssaro4^K220L^*, *PIR1* promoter, and *TEF1* terminator regions were amplified from *S. stipitis* NBRC 10063 genomic DNA by PCR using Overlap-Aro4-F/R, PIR1p-F/R and TEF1t-F/R primers, respectively. These fragments were joined by standard overlap PCR to obtain the *aro4_K220L_* expression cassette (*PIR1p*-*aro4_K220L_*-*TEF1t*). Site-directed mutagenesis was performed by overlap PCR (Ho et al., 1989) using Overlap-Aro4_upstr_mut-R and Overlap-Aro4_dwnstr_mut-F primers to introduce the corresponding mutations into *ARO4*. The constructed expression cassette (*PIR1p- aro4_K220L_ -TEF1t*) was cloned into pCU5. Inverse PCR with the Inv-pCU5_pInU5-aro4m-F/R primers was performed to replace *ARS2* and *CEN6* of pCU5 to the constructed expression cassette. These fragments were ligated by the In-Fusion method. The resulting plasmid is designated by pInU5-ARO4m.

To construct the overexpression cassette of *Ssaro7_G139S_* gene, DNA fragments of *Ssaro7_G139S_*, *ENO1* promoter, and *UAG* terminator regions were amplified from *S. stipitis* NBRC 10063 genomic DNA by PCR using Overlap-Aro7-F/R, ENO1p-F/R and UAGt-F/R primers, respectively. These fragments were joined by standard overlap PCR to obtain the *aro7_G139S_* expression cassette (*ENO1p*-*aro7_G139S_*-*UAGt*). Site-directed mutagenesis was performed by overlap PCR (Ho et al., 1989) using Overlap-Aro7_upstr_mut-R and Overlap-Aro7_dwnstr_mut-F primers to introduce the corresponding mutations into *ARO7*.

The constructed expression cassette (*ENO1p-aro7_G139S_-UAGt*) was cloned into pCU5. Inverse PCR with the Inv-pCU5_pInU5-aro7m-F/R primers was performed to replace *ARS2* and *CEN6* of pCU5 to the constructed expression cassette. These fragments were ligated by the In-Fusion method. The resulting plasmid is designated by pInU5-ARO7m.

To construct the co-overexpression cassette of *Ssaro4_K220L_* and *Ssaro7_G139S_* genes, DNA fragment of *aro7_G139S_* expression cassette (*ENO1p*-*aro7_G139S_*-*UAGt*) was amplified from pInU5-ARO4m by PCR using PIRp-F/TEF1t-R. The amplified expression cassette was cloned into pInU5-ARO4m. Inverse PCR with the Inv-pInU5-aro4m-F/R primers was performed to insert the constructed expression cassette. These fragments were ligated by the In-Fusion method. The resulting plasmid is designated by pInU5-ARO47m. Inverse PCR with the Inv-pInU5-aro4m-delt_aro4-F and PIRp-R primers was performed to remove the aro4K230L-cording region of pInU5-ARO4m. This fragment was self-ligated by the In-Fusion method, and the resulting plasmid was designated by pInU5-em.

**Supplementary Table 1 | Primers used in this study.**

| Primers | Sequence |
| --- | --- |
| SsCEN500-F | aagatctcagaagcattcatagtgtcttacgtg |
| SsCEN500-R | atggaaatgtcaaatctgaattcaatatagccgac |
| SsARS2-F | catgtactgaattcagtataggatatgg |
| SsARS2-R | tgcttctgagatcttctgcggtgtctac |
| Ec-element-F | atttgacatttccataggctccgccc |
| Ec-element-R | ccggatccggtggcacttttcgggg |
| IF-URA5-F | gtgccaccattagtggaaggaaagaaatcc |
| IF-URA5-R | ctgaattcagtacatgattgatttc |
| PIRp-F | aaggctctttgaatttactttgcc |
| PIRp-R | tgtaaatcaatcaggtttattg |
| GLN1t-F | atgtctggctggtttcc |
| GLN1t-R | taacgaataatgaacattagtaccac |
| cas9_opt_stipitis-F | cctgattgatttacaatggctccaaagaagaagagaaagg |
| cas9_opt_stipitis-R | cgtaaattaatcagcttacacttttctcttcttctttggtcc |
| Int-cas9_pCU5-F | aataggggttccgcgaaggctctttgaatttactttgcc |
| Int-cas9_pCU5-R | tttccttccactaattaacgaataatgaacattag |
| IF-SNR52p-F | gttcattattcgttacatgagaagaagagaaattgttc |
| IF-SNR52p-R | acaacactttgtagtgaaataaatgtatcttgttgg |
| gRNA_ade2_SUP4ter-F | actacaaagtgttgtaccatg |
| SUP4ter-R | tttccttccactaatagacataaaaaacaaaaaaacaccg |
| Inv-pCU5-cas_gRNA-F | attagtggaaggaaagaaatccg |
| Inv-pCU5-cas_gRNA-R | taacgaataatgaacattagtaccac |
| Int-Ade2_pCU5-F | acagcagctatgggaaagtc |
| Int-Ade2_pCU5-R | ttcaaactttcacttatctttg |
| Inv-pCU5-F | aagtgaaagtttgaatttccataggctccgcccccc |
| Inv-pCU5-R | tcccatagctgctgtcaatcttggggtagattccg |
| TEF1t-F | gctgattaatttacgtatattcag |
| TEF1t-R | gtgctgatgtagtgatc |
| Overlap-TAL1-F | cctgattgatttacaatgtccaccaccttgatcttg |
| Overlap-TAL1-R | cgtaaattaatcagcctatctgaacaagatgatgg |
| Inv-pInA2-TAL1-F | tcactacatcagcacacagcagctatgggaaagtc |
| Inv-pInA2-TAL1-R | aattcaaagagccttcgcggaacccctatttgtttatttttc |
| ENO1p-F | tacatcaccagccaccg |
| ENO1p-R | tgttagtgcgggaatcg |
| UAGt-F | ttttatttttcctttccttaaatc |
| UAGt-R | caatcttggggtagattccg |
| Overlap-4CL2-F | attcccgcactaacaatgactactcaggacgtcatc |
| Overlap-4CL2-R | aaaggaaaaataaaactagttcatcaagccgttgg |
| TEF1p-F | ttcatgaagtacgataagg |
| TEF1p-R | tgtagatagacttagattg |
| Overlap-VST1-F | catacaatttccatcatggcctctgtcgaagaattc |
| Overlap-VST1-R | aaaccagccagacatctagttggtgacagttgggac |
| Inv-pInA2-TAL_pInA2-T4V-F | gttcattattcgttaacagcagctatgggaaagtc |
| Inv-pInA2-TAL_pInA2-T4V-R | gtggctggtgatgtagtgctgatgtagtgatcagaaatg |
| Overlap-Aro4-F | cctgattgatttacaatgtcccaaacaccagtac |
| Overlap-Aro4_upstr_mut-R | gttaccaagggtggtagtgatggcag |
| Overlap-Aro4_dwnstr_mut-F | accacccttggtaacgacaactgtttc |
| Overlap-Aro4-R | cgtaaattaatcagcttatgccttgagggctc |
| Inv-pCU5_pInU5-aro4m-F | tcactacatcagcacattagtggaaggaaagaaatcc |
| Inv-pCU5_pInU5-aro4m-R | aattcaaagagccttcgcggaacccctatttgtttatttttc |
| Overlap-Aro7-F | attcccgcactaacaatggatttcacaaagccc |
| Overlap-Aro7_upstr_mut-R | aaccgaagacaagttctcctgttgctc |
| Overlap-Aro7_dwnstr_mut-F | aacttgtcttcggttctgactcaggac |
| Overlap-Aro7-R | aaaggaaaaataaaactagacgtatttttcaatctg |
| Inv-pCU5_pInU5-aro7m-F | tctaccccaagattgacagcagctatgggaaagtc |
| Inv-pCU5_pInU5-aro7m-R | gtggctggtgatgtacgcggaacccctatttgtttatttttc |
| Inv-pInU5-aro4m-F | tctaccccaagattgattagtggaaggaaagaaatcc |
| Inv-pInU5-aro4m-R | gtggctggtgatgtaggtggcacttttcggggaaatgtg |
| Inv-pInU5-aro4m-delt_aro4-F | cctgattgatttacagctgattaatttacgtatattc |
| qRT-PCR_ARO4-F | tccagcacgaaatcaaagcc |
| qRT-PCR_ARO4-R | ggctctgtcgtcctttccag |
| qRT-PCR_ARO7-F | tgtagccgaagccaagtacc |
| qRT-PCR_ARO7-R | ctacggcgctgtttgtgatg |


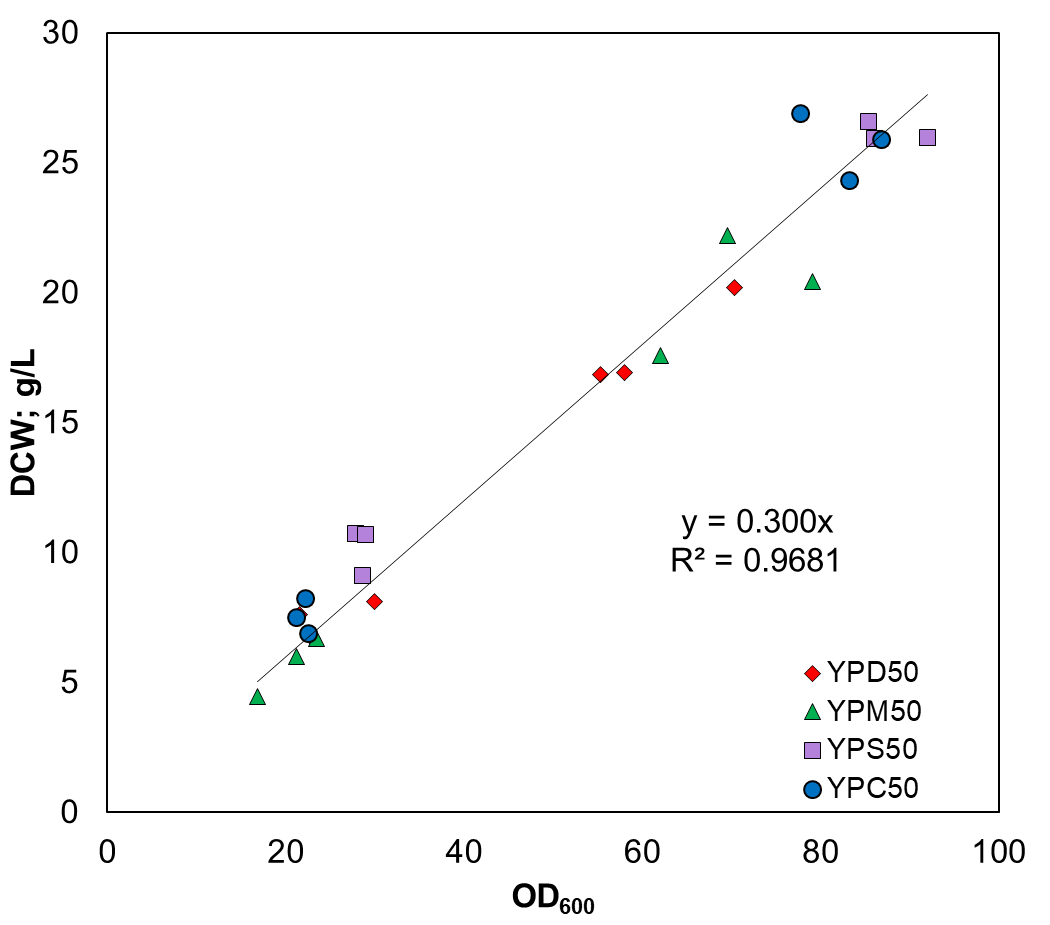


**Fig. S1 The calibration curve between dry cell weight (DCW; g/L) and the value of OD_600_**. Yeast cells harvested from YPD50, YPC50, YPM50, and YPS50 medium after 24 h and 48 h fermentations were collected for measuring the values of OD_600_ and dry cell weight (DCW; g/L).


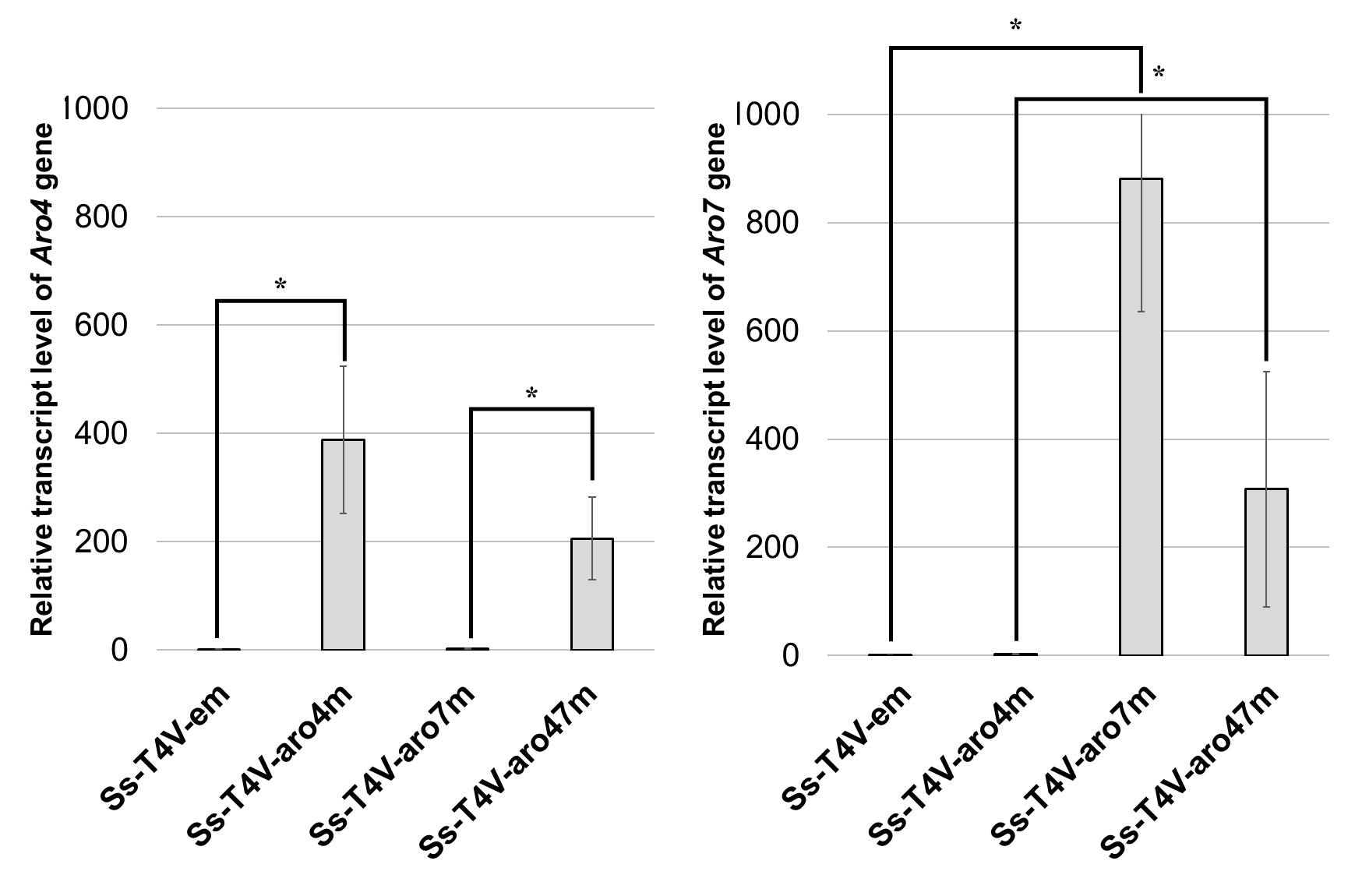


**Fig. S2 Transcript levels for genes overexpressed in recombinant strains after fermenting YPD50 medium for 48 h.** The relative transcript level of each gene is shown as a fold change in the mRNA level from the average of control strain. Data are presented as the means ± standard deviation (n = 3). Statistical significance was determined by one-way ANOVA with Tukey's range test (*; *p* < 0.05).


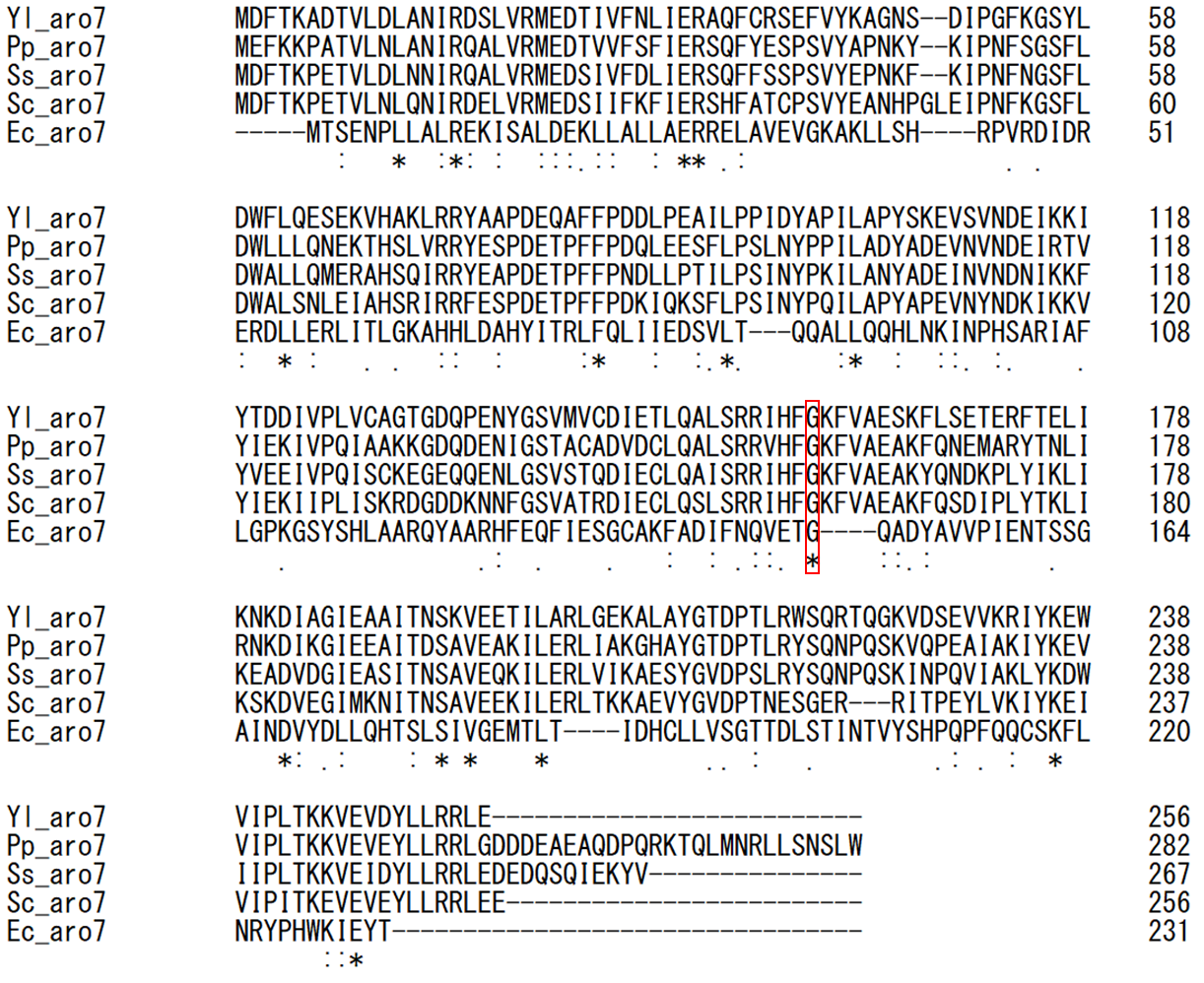


**Fig. S3 Alignment of amino acid sequence of chorismate mutase (Aro7p) from *S. stipitis* (Ss_aro7), *Y. lipolytica* (Yl_aro7), *Pichia pastoris* (Pp_aro7), *S. cerevisiae* (Sc_aro7), and *Escherichia coli* (Ec_aro7) performed by using ClustalW.** Identical residues in all five proteins are indicated by asteriscs below the sequence. The residue suggested to be involved in feedback inhibition is shown in red square.


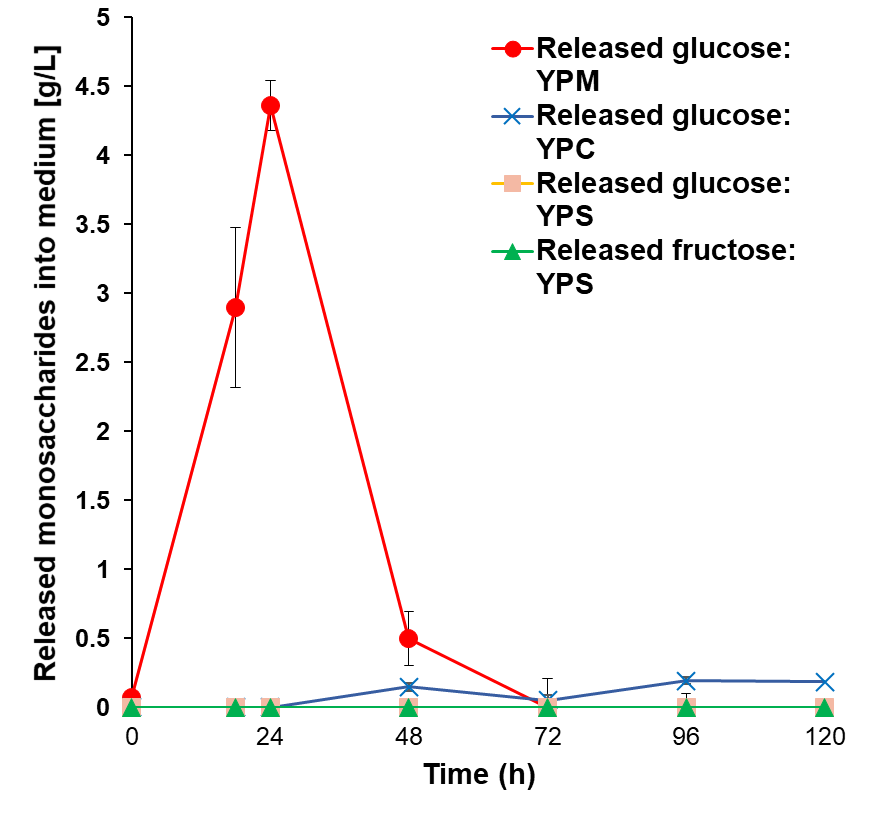


**Fig. S4 Time course of accumulation of monosaccharides released from disaccharides hydrolysis during fermentation**. Data are presented as the means ± standard deviation (n = 3).

**
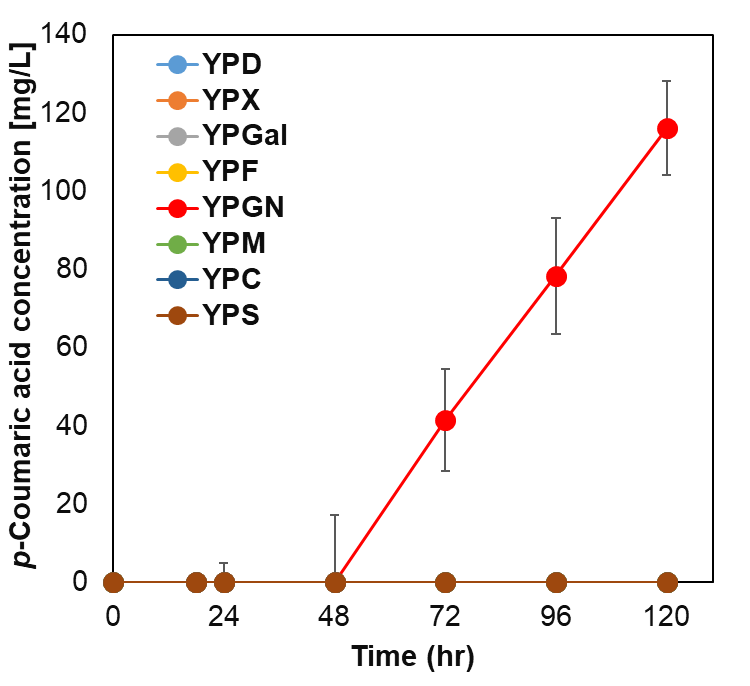
**

**Fig. S5 Time course of accumulation of *p*-coumaric acid during fermentation**. Data are presented as the means ± standard deviation (n = 3).

**
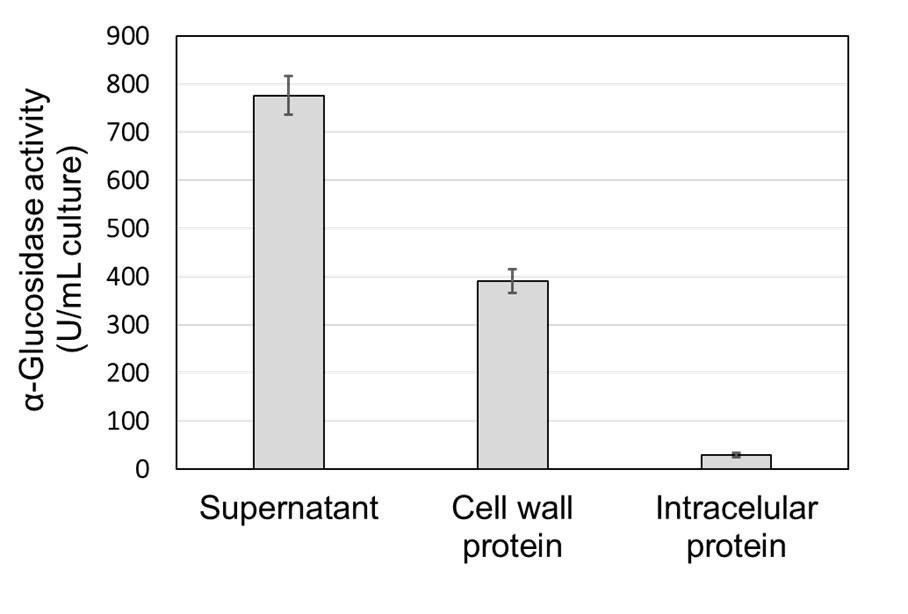
Fig. S6 The specific activity of α-glucosidase of protein samples extracted from the culture broths in the fermentation with maltose.** The specific activities were normalized by the original volume of the culture medium. Data are presented as the means ± standard deviation (n = 3).

**References in the Supplemental data**

Cao, M., Seetharam, A.S., Severin, A.J., Shao, Z., 2017. Rapid Isolation of Centromeres from *Scheffersomyces stipitis*. ACS Synth. Biol. 6, 2028–2034. https://doi.org/10.1021/acssynbio.7b00166

Ho, S.N., Hunt, H.D., Horton, R.M., Pullen, J.K., Pease, L.R., 1989. Site-directed mutagenesis by overlap extension using the polymerase chain reaction. Gene 77, 51–59. https://doi.org/10.1016/0378-1119(89)90358-2

Yang, V.W., Marks, J.A., Davis, B.P., Jeffries, T.W., 1994. High-efficiency transformation of *Pichia stipitis* based on its *URA3* gene and a homologous autonomous replication sequence, *ARS2*. Appl. Environ. Microbiol. 60, 4245–4254. https://doi.org/10.1128/aem.60.12.4245-4254.1994
